# Perfluorinated Compounds (PFCs) in River Waters of Central Italy: Monthly Variation and Ecological Risk Assessment (ERA)

**DOI:** 10.1007/s00244-023-00993-4

**Published:** 2023-04-06

**Authors:** Federica Castellani, Mara Galletti, Fedra Charavgis, Alessandra Cingolani, Sonia Renzi, Mirko Nucci, Carmela Protano, Matteo Vitali

**Affiliations:** 1 Department of Public Health and Infectious Diseases, University of Rome la Sapienza, P.le Aldo Moro, 5, 00185 Rome, Italy; 2 Department of Ecological and Biological Sciences, Tuscia University, Largo dell’Università snc, 01100 Viterbo, Italy; 3 ARPA Umbria, Via Carlo Alberto Dalla Chiesa, 23, 05100 Terni, Italy; 4 ARPA Umbria, Via Pievaiola 207/B-3, 06132 Perugia, Italy

## Abstract

Perfluorinated compounds (PFCs) are a wide class of emerging pollutants. In this study, we applied the US EPA method 533 for the determination of 21 PFCs in river water samples. In particular, this method was used to investigate the presence of the target PFCs in six rivers in central Italy during a 4-month-long monitoring campaign. In 73% of the analyzed samples, at least some of the target PFCs were detected at concentrations higher than the limit of detection (LOD). The sum of the 21 target analytes (∑_21_PFCs) ranged from 4.3 to 68.5 ng L^−1^, with the highest concentrations measured in the month of June, probably due to a minor river streamflow occurring in the warmer summer months. Considering the individual congeners, PFBA and PFPeA, followed by PFHxA and PFOA, were the predominantly detected compounds. Short- and medium-chain PFCs (C4–C9) prevail over the long-chain PFCs (C10–C18), likely due to the increased industrial use and the higher solubility of short-chain PFCs compared to long-chain PFCs. The ecological risk assessment, conducted by using the risk quotient method, highlighted that the risk for aquatic environments associated with PFBA, PFPeA, PFBS, PFHxA and PFOA was low or negligible. Only for PFOA, there was a medium level of risk in two rivers in the month of June. With regard to PFOS, 54% of the river water samples were classified as “high risk” for the aquatic environment. The remaining 46% of the samples were classified as “medium risk.”

**Supplementary Information:**

The online version contains supplementary material available at 10.1007/s00244-023-00993-4.

Perfluorinated compounds (PFCs) are a wide class of organic substances characterized by a fluorinated hydrophobic carbon chain, bound to a hydrophilic head group (González-Barreiro et al. 2006; Kancharla et al. 2022). Because of their chemical structure, PFCs present unique properties such as surface activity, thermal and acid resistance and repellency of both water and oil that make them ideal for several commercial and industrial applications (Wang et al. 2017). Since 1960s, PFCs were used as constituents of a wide range of products including fluoropolymers (e.g., polytetrafluoroethylene; PTFE), liquid repellents for paper, food packaging, cookware, textiles, leather and carpets, and as firefighting foams (Wang et al. 2009; Blaine et al. 2013; Ojemaye and Petrik 2019; Kurwadkar et al. 2022). Due to the strength of carbon–fluorine (C–F) bonds, PFCs are extremely resistant to any degradation process, such as hydrolysis, photolysis, metabolism and biodegradation (Li et al. 2012; Organisation for Economic Co-operation and Development 2018). This property, together with their water solubility and the capacity to bioaccumulate and to biomagnify, has determined their ubiquitous distribution in the environment, wildlife and their presence in human biological samples across the world (Butt et al. 2010; Zhang et al. 2014; Campo et al. 2016; Lam et al. 2017; Sedlak et al. 2017; Boisvert et al. 2019). Among PFCs, perfluorooctane sulfonate (PFOS) and perfluorooctanoic acid (PFOA) have been the most widely studied (Post et al. 2012; Li et al. 2013; Miralles-Marco and Harrad 2015; Liang et al. 2022). In 2009, PFOS and its salts were included in the Stockholm convention as new persistent organic pollutants (new POPs) due to their persistence, ability to bioaccumulate and toxicological concern (UNEP 2009; Domingo 2012). With regard to PFOA, its manufacture and use were initially phased out by the manufacturer to reduce global emissions (USEPA 2010, 2012), and in 2019, PFOA, its salts and PFOA-related compounds are listed in Annex A of the Stockholm convention (UNEP 2019). However, due to resistance to degradation and chemical stability characterizing this class of compounds, human exposure and environmental contamination are expected to continue for the near future and beyond (Lindrom et al. 2011; Post et al. 2012; Ahmed et al. 2020). In recent years, the human and environmental health concerns associated with this class of compounds have pushed the scientific community to focus their attention on the presence of PFCs in environmental matrices and, in particular, in the water matrix (Thompson et al. 2011; Barreca et al. 2018; Deng et al. 2019; Zhang et al. 2019, 2021; Fauconier et al. 2020). Indeed, due to the high hydrophilicity of PFCs, the water environment is an important reservoir of these compounds and the main pathway through which PFCs undergo long-range transport (Sungur 2022). The presence of these compounds in the water environment can cause adverse effects on aquatic flora and fauna. In particular, scientific evidence demonstrated that PFCs can inhibit algae growth, induce cytotoxic and genotoxic effects on invertebrates and fishes and cause serious negative outcomes on amphibians (Latała et al. 2009; Liu et al. 2014; Kim et al. 2015; Ayanda et al. 2018; Savoca and Pace 2021). In Italy, the analytical determination of these substances in water samples started in 2013, after the discovery of a massive groundwater contamination in a vast area in northern Italy (Valsecchi et al. 2015; Bonelli et al. 2020; Chiesa et al. 2022). Although the nature of these pollutants does not exclude large-scale distribution, the determination of PFAs was carried out only in the surface waters of northern Italy. In the present study, US EPA method 533 was tested and applied for the determination of 21 PFCs (C4–C14, C16 and C18 perfluoroalkyl carboxylic acids and C4–C10 and C12 perfluoroalkyl sulfonic acids). Being an exploratory monitoring campaign, the target compounds were selected to investigate the presence of compounds with different carbon chain lengths. The tested method was then applied to investigate the presence of target PFCs in river water samples collected in Umbria region (central Italy) during a four-month monitoring campaign (March–June 2022). To our knowledge, this is the first study investigating the presence of PFCs in Umbria region (central Italy). In addition, the monitoring campaign conducted over several months allowed an investigation of the temporal trends of these pollutants.

## Materials and Method

### Sample Collection

**Fig. 1:**
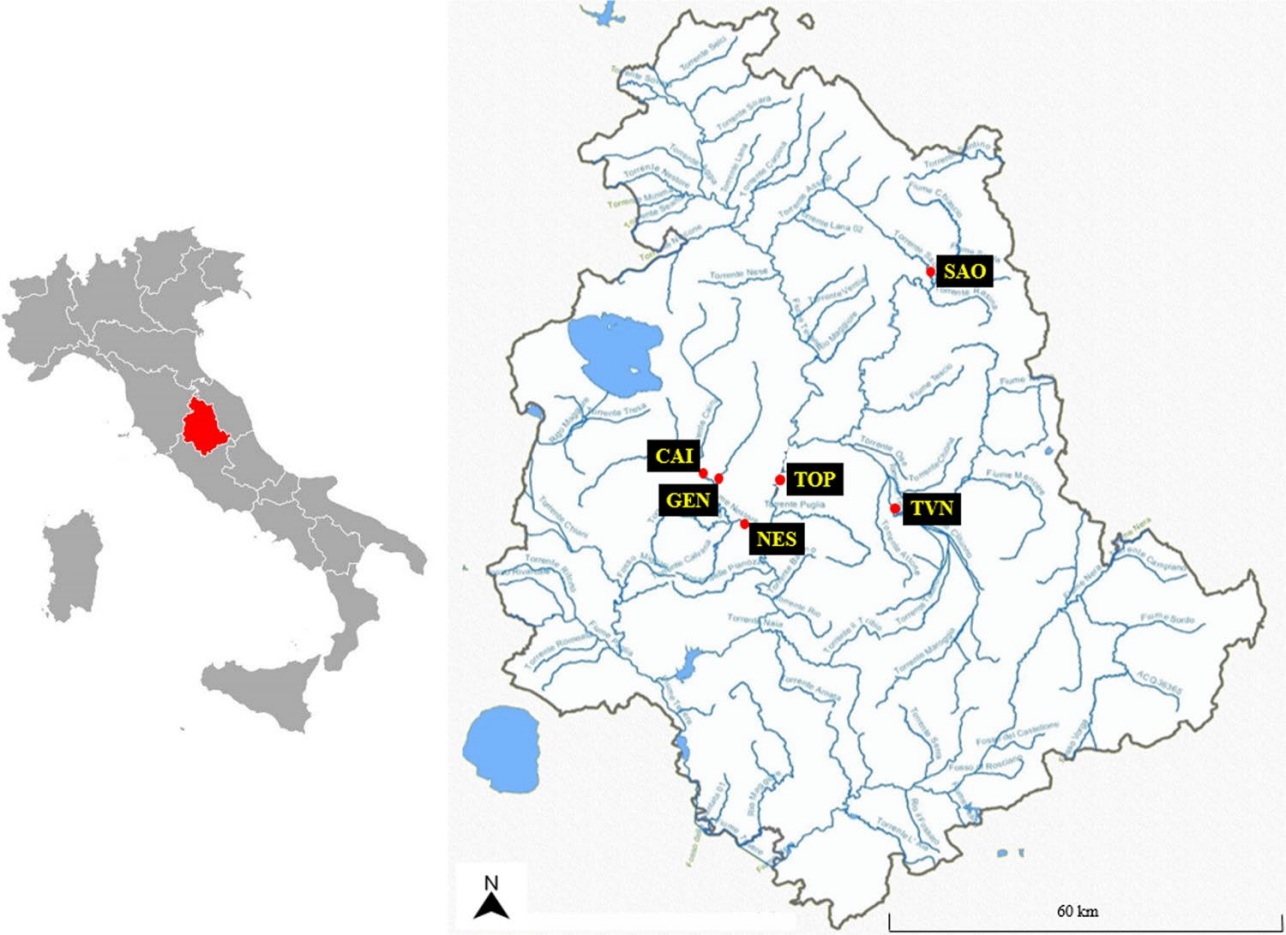
Geographical distribution of the 6 sampling sites in Umbria region (central Italy)

From March to June 2022, grab samples of surface water were collected from six different rivers in the central–northern area of Umbria region in central Italy (Fig. 1). River waters were collected as grab samples because, given the surfactant properties of these compounds, US EPA (Environmental Protection Agency) does not recommend composite sampling (US EPA 2022). The choice of the most appropriate monitoring sites was made on the basis of a previous study conducted by the ARPA Umbria regional agency for environmental protection, as reported by Nucci et al. (2019a, b) and by Charavgis et al. (2022). The results obtained from this previous investigation highlighted the necessity of monthly monitoring of PFCs, both in rivers where the concentrations of these substances exceeded the maximum levels fixed by directive 2013/39/EU (European Commission 2013) and in those rivers affected by significant sources of urban, agricultural and industrial pollution, which are NES, CAI, GEN, TVN rivers identified in Table 1. Details on sampling dates, locations and area description of the investigated rivers are shown in Table 1. All the grab samples were stored in a 1-L polypropylene (PP) tube, pre-cleaned with methanol followed by ultrapure water in order to avoid contaminations. The samples were stored in a cool bag, transported to the laboratory and stored at + 4 °C until analysis.

**Table 1:** Sampling locations and date, area description and major type of contamination

	Site	Sampling dates	Longitude	Latitude	Area description	Type of contamination
CAI_mar CAI_apr CAI_may CAI_jun	Caina	03/03/2022 04/05/2022 05/10/2022 06/03/2022	12° 15′ 44.73″ E	43° 0′ 9.06″ N	Rural area	Water treatment plants, urban waste water and agro-livestock
NES_mar NES_apr NES_may NES_jun	Nestore	03/03/2022 04/05/2022 05/10/2022 06/03/2022	12° 21′ 58.61″ E	42° 54′ 27.60″ N	Urban area/rural area	Water treatment plants, urban waste water and agro-livestock
GEN_mar GEN_apr GEN_may GEN_jun	Genna	03/03/2022 04/05/2022 05/11/2022 06/04/2022	12° 17′ 29.24″ E	42° 58′ 8.93″ N	Rural area	Water treatment plants, urban waste water and agro-livestock
TOP_mar TOP_apr TOP_may TOP_jun	Topino	03/02/2022 04/05/2022 05/11/2022 06/04/2022	12° 30′ 27.33″ E	43° 1′ 34.51″ N	Urban area/rural area	Industrial plants, water treatment plants and urban waste water
SAO_mar SAO_apr SAO_may SAO_jun	Saonda	03/07/2022 04/05/2022 05/12/2022 06/05/2022	12° 39′ 25.20″ E	43° 15′ 45.79″ N	Rural area	Industrial plants, urban waste water and agro-livestock
TVN_mar TVN_apr TVN_may TVN_jun	Timia	03/07/2022 04/05/2022 05/12/2022 06/05/2022	12° 36′ 38.53″ E	42° 55′ 50.37″ N	Urban area/industrial area	Industrial plants, water treatment plants and urban waste water

### Chemicals and Reagents

Methanol (MeOH) LC–MS grade was supplied by Merck (Darmstadt, Germany), and ultrapure water was obtained from a Milli-Q filter system (Millipore, Bedford, MA, USA). Ammonium acetate, HPLC grade, was from Merck (Darmstadt, Germany). Stock standards containing 2 µg mL^−1^ of the target analytes (Table 2) were obtained from Wellington Laboratories Inc. (Guelph, Ontario, Canada). Mass-labeled injection standards (IS; Table 2) at a concentration of 2 µg mL^−1^ were purchased from Wellington Laboratories Inc. (Guelph, Ontario, Canada). Mass-labeled extraction standards (ES; Table 2) at a concentration of 2 µg mL^−1^ were purchased from Wellington Laboratories Inc. (Guelph, Ontario, Canada). Stock solutions (20 ng mL^−1^) of the analytes were prepared in MeOH:H_2_O (80:20, v/v), in polypropylene (PP) volumetric tubes, and then stored at + 4 °C. Water–methanol (80:20, v/v) calibration solutions at concentrations 0.2, 1.0, 10 and 20 ng mL^−1^ were freshly prepared before each measurement and stored at + 4 °C. The long-term stability of stocks was monitored to guarantee the consistency of standards. For the verification of analytical precision and accuracy, a certified reference material (CRM) IRMM-428 for PFCs in water obtained from the European Commission Joint Research Centre, Institute for Reference Materials and Measurements (IRMM), was analyzed.

**Table 2:** Internal standards, retention times (RT) in minutes, limit of detection (LOD, ng L^−1^), limit of quantification (LOQ, ng L^−1^), precursor and product ions (q: qualifier, Q: quantifier) of the target analytes

Analytes	Acronym	Internal Standard	RT (min)	LOD	LOQ	Precursor Ion (m/z)		Product Ion (m/z)
Perfluorobutanoic acid	PFBA	M3PFBA	0.81	0.48	5	213		169
Perfluoropentanoic acid	PFPeA	M5PFPeA	1.98	0.05	0.65	263		219
Potassium perfluoro-1-butanesulfonate	PFBS	M3PFBS	2.08	0.04	0.65	298.9	Q	80
							q	98.9
Perfluorohexanoic acid	PFHxA	M5PFHxA	3.56	0.06	0.65	312.9		268.9
Sodium perfluoro-1-pentanesulfonate	PFPeS	M3PFHxS	3.82	0.002	0.65	349	Q	80
							q	99
Perfluoroheptanoic acid	PFHpA	M4PFHpA	5	0.03	0.65	362.9	Q	319
							q	169
Sodium perfluoro-1-hexanesulfonate	PFHxS	M3PFHxS	5.13	0.02	0.65	398.9	Q	80
							q	99
Perfluorooctanoic acid	PFOA	M8PFOA	6.1	0.11	5	412.9	Q	368.9
							q	169
Sodium perfluoro-1-heptanesulfonate	PFHpS	M8PFOS	6.17	0.005	0.65	449	Q	80
							q	99
Perfluorononanoic acid	PFNA	M9PFNA	7.09	0.04	0.65	462.9	Q	418.9
							q	169
Sodium perfluoro-1-octanesulfonate	PFOS	M8PFOS	7.09	0.03	0.2	498.9	Q	80
							q	99
Perfluorodecanoic acid	PFDA	M6PFDA	7.74	0.05	0.65	512.9	Q	469
							q	169
Sodium perfluoro-1-nonanesulfonate	PFNS	M8PFOS	7.65	0.04	0.65	549	Q	80
							q	99
Perfluoroundecanoic acid	PFUdA	M7PFUdA	8.45	0.07	0.65	562.9	Q	519
							q	169
Sodium perfluoro-1-decanesulfonate	PFDS	M8PFOS	8.38	0.05	0.65	598.9	Q	80
							q	99
Perfluorododecanoic acid	PFDoA	MPFDoA	9.04	0.09	0.65	612.9	Q	569
							q	169
Perfluorotridecanoic acid	PFTriA	M2PFTeDA	9.56	0.07	0.65	662.9	Q	619
							q	169
Sodium perfluoro-1-dodecanesulfonate	PFDoS	M8PFOS	9.42	0.05	0.65	699	Q	80
							q	99
Perfluorotetradecanoic acid	PFTeA	M2PFTeDA	10.08	0.06	0.65	712.9	Q	669
							q	169
Perfluorohexadecanoic acid	PFHxDA	M2PFTeDA	11.92	0.21	0.65	813	Q	769
							q	169
Perfluorooctadecanoic acid	PFODA	M2PFTeDA	13.38	0.20	0.65	913	Q	869
							q	169
*Mass-labeled extraction standards*								
Perfluoro-N-(^13^C_4_)butanoic acid	MPFBA		0.89			217		172
Perfluoro-N-(^13^C_5_)pentanoic acid	M5PFPeA		1.85			268		223
Sodium perfluoro-1-(2,3,4-^13^C_3_)butanesulfonate	M3PFBS		2.13			302		80
Perfluoro-N-(1,2,3,4,6-^13^C_5_)hexanoic acid	M5PFHxA		3.43			318		273
Perfluoro-N-(1,2,3,4-^13^C_4_)heptanoic acid	M4PFHpA		4.83			367		322
Odium perfluoro-1-(1,2,3-^13^C_3_)hexanesulfonate	M3PFHxS		4.96			402		80
Perfluoro-N-(^13^C_8_)octanoic acid	M8PFOA		5.96			421		376
Perfluoro-N-(^13^C_9_)nonanoic acid	M9PFNA		6.87			472		427
Sodium perfluoro-1-(^13^C_8_)octanesulfonate	M8PFOS		6.9			507		80
Perfluoro-N-(1,2,3,4,5,6-^13^C_6_)decanoic acid	M6PFDA		7.64			518.9		473.9
Perfluoro-N-(1,2,3,4,5,6,7-^13^C_7_)undecanoic acid	M7PFUdA		8.3			570		525
Perfluoro-N-(1,2-^13^C_2_)dodecanoic acid	MPFDoA		8.86			614.9		570
Perfluoro-N-(1,2-^13^C_2_)tetradecanoic acid	M2PFTeDA		9.92			715		670
*Mass-labeled injection standards*								
Perfluoro-N-(2,3,4-^13^C_3_)butanoic acid	M3PFBA		0.9			216		172
Perfluoro-N-(1,2-^13^C_2_) octanoic acid	M2PFOA		5.96			415		370
Sodium perfluoro-1-(1,2,3,4-^13^C_4_) octane Sulfonate	MPFOS		6.9			503		99
Perfluoro-N-(1,2-^13^C_2_) decanoic acid	MPFDA		7.65			515		470

### Samples Extraction and Instrumental Analysis

The extraction of the aqueous samples was performed as described in US EPA Method 533 (US EPA 2019) with minor modifications. Briefly, an aliquot of 250 mL of the water sample was collected in a 250-mL high-density polypropylene (HDPE) bottle with a narrow neck. The aliquot was spiked with 250 µL of the ES at the concentration of 20 ng mL^−1^ and intensively mixed with a vortex mixer. The sample was then extracted via solid phase extraction (SPE) using Strata™-XL-AW cartridge (100 mg, 6 mL, Phenomenex, CA, United States). The cartridge was previously conditioned with 10 mL of MeOH followed by 10 mL of phosphate buffer 0.1 M. The sample was then passed through the cartridge with the aid of a membrane pump at a flow rate of 5 mL min^−1^ and cleaned with 10 mL of ammonium acetate (1 g L^−1^) followed by 1 mL of MeOH. Subsequently, the cartridge was dried for 5 min under high vacuum (15–20 mmHg). The target compounds were than eluted with 2 × 5 mL MeOH:NH_4_OH and dried under a gentle flux of nitrogen (purity > 99.999%). The sample was then re-suspended in 250 μL of IS solution (20 ng mL^−1^) and analyzed by high-pressure liquid chromatography and tandem mass spectrometry with electrospray ionization (HPLC–ESI–MS/MS). Blanks (Milli-Q H_2_O), fortified with ES solution at the same concentration as the samples, were prepared and analyzed simultaneously. Chromatographic separations were performed using an Agilent 1290 Infinity II HPLC (Agilent Technologies, Santa Clara, CA, USA), fitted with an Agilent 1260 G7129A autosampler. The HPLC was connected to an Agilent 6475 triple quadrupole mass spectrometer with a Jet Stream 6450 electrospray ionization unit (AJS-ESI) supplied by Agilent. The chromatographic column was a Zorbax Eclipse Plus C18 RRHD (50 × 3.0 mm, 1.8 μm) purchased from Agilent Technologies (Santa Clara, CA). A guard column (Zorbax Eclipse Plus C18 RRHD, 4.6 × 30 mm, Agilent Technologies, USA) was installed between the solvent mixer and injector module to avoid instrumental contamination.

### Chromatographic and Mass Spectrometric Conditions

**Fig. 2:**
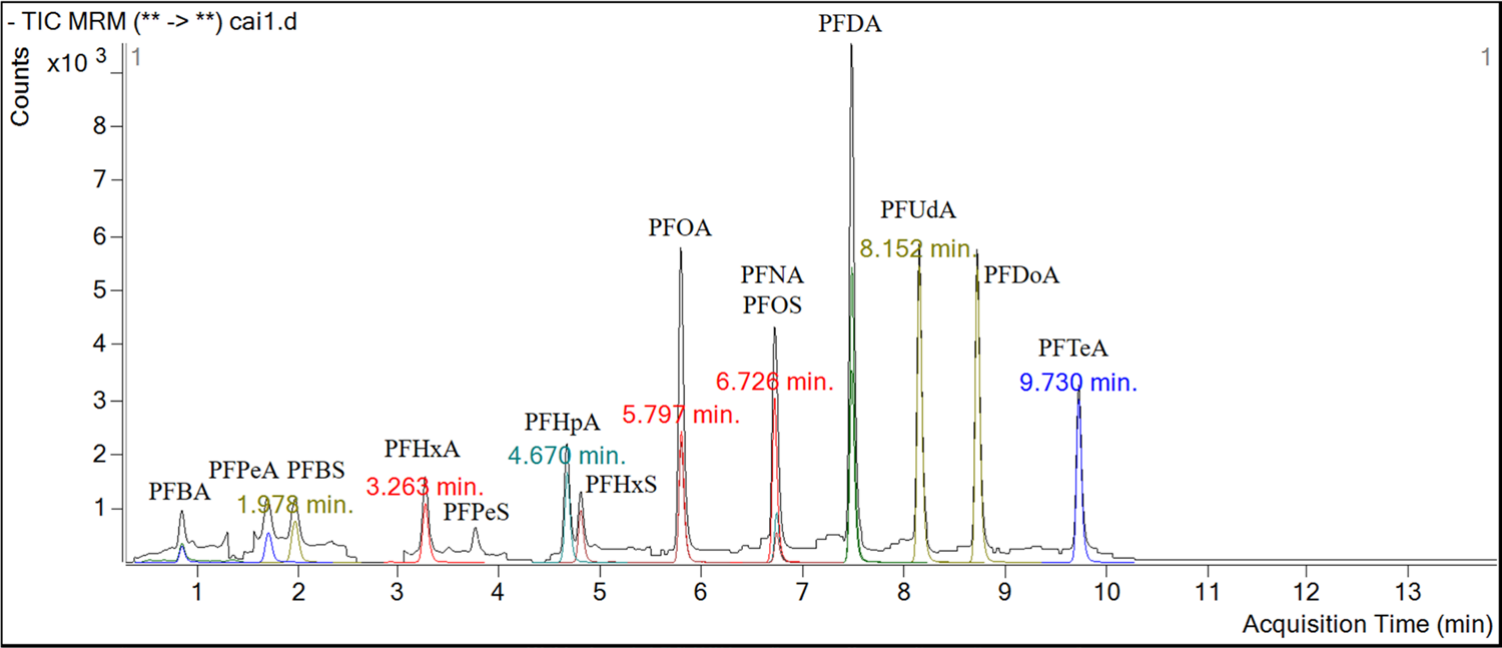
HPLC-AJS-ESI–MS/MS total ion current (TIC), acquired in MRM mode, of CAI river water sampled in the month of June

Chromatography was performed using H_2_O (A) and MeOH (B) both containing 2 mM of ammonium acetate at a flow rate of 500 µL min^−1^. Gradient elution started at 40% of B for 0.5 min and was raised to 80% within 7.5 min; after 4 min in isocratic condition, mobile phase B was raised to 95% and equilibrated for 1 min. The initial conditions were then restored, and the system was equilibrated for 2 min. The column temperature was set at 40 °C, and the injection volume was 5 μL. The retention times (RTs) of the analytes are listed in Table 2. AJS-ESI–MS/MS measurements were taken in negative ion mode using multiple reaction monitoring (MRM). Mass spectrometer parameters were obtained by tuning the electrical parameters for each compound by infusion of standard solutions at concentration of 1 μg mL^−1^ at flow rate of 0.7 μL min^−1^. The source gas temperature and the sheath gas temperature were set at 320 °C and 350 °C, respectively. The ion capillary (IS) and the nozzle voltage were 3750 V and 1500 V, respectively. The gas flow and the sheath gas flow were set at 5 L min^−1^ and 12 L min^−1^. The nebulizer was 50 psi, and the cell accelerator voltage was 7 V for all the analytes under study. The collision energies were between 5 and 95 eV, and the fragmentor values were between 60 and 210 V. Precursor and product ions and the mass-labeled compound chosen as internal standard are reported in Table 2. Figure 2 shows a HPLC–MS/MS total ion current (TIC) chromatogram obtained by injecting 5 μL of CAI river water sampled in the month of June.

### Quality Control/Quality Assurance

The US EPA method 533 was tested by studying repeatability, reproducibility, specificity, sensitivity and recovery. For the quantification of the samples, the isotope dilution method was applied. A total of 13 mass-labeled compounds (ES) were used as surrogate standards with the purpose to determine the relative response factor (RRF) of the related native compound and to verify the retention time (RT). For the native compounds without the corresponding ES, the one with the most suitable structure was chosen (Table 2). Repeatability and reproducibility were evaluated on seven independent tests, by spiking different aliquots of the same river water sample at different concentrations (0.2, 0.65, 5, 10 and 20 ng L^−1^). All the tests were performed in sevenfold. A five-point calibration curve covering concentrations from 0.2 to 20 ng mL^−1^ was used for the quantification of PFASs in environmental samples. The limits of quantification (LOQs) of each analyte were defined as the concentration value equal or below a value of 30% of the relevant environmental quality standards (EQS; 1000 ng L^−1^ for PFHxA, 3000 ng L^−1^ for PFPeA and PFBS, 7000 ng L^−1^ for PFBA, 100 ng L^−1^ for PFOA and 0.65 ng L^−1^ for PFOS) as established by Commission Directive 2009/90/EC. The method recoveries were firstly assessed by analyzing water samples spiked with target analytes at different concentrations (0.2, 0.65, 5, 10 and 20 ng L^−1^). Even in this case, all the tests were performed in sevenfold. Moreover, recoveries were assessed by spiking every sample with ES solution at the concentration of 20 ng mL^−1^. Quality control (QC) standards (one procedural blank sample and one calibration standard at the concentration of 1 ng mL^−1^) were analyzed every tenth sample, in order to control the instrument sensitivity. The ion ratio between the qualitative MS/MS transition and the quantitative MS/MS transition response and the RT were recorded for each analyte and every sample, in order to correctly identify the compounds. The performance of the instrument was also monitored by adding IS at 20 ng mL^−1^ to the samples just before the injection. The method reliability was also examined by analyzing the certified reference material IRMM-428 (tap water).

### Statistical Analysis

Statistical analysis was carried out by using the statistical software R (R-project for statistical computing, version 3.0, 32-bit). Principal component analysis (PCA) was performed on the mean concentrations of PFCs in order to cluster the tracers of the main emission sources. Before performing the PCA, the matrix of the data was transformed by column mean centering and row and column autoscaling to correct for different variable scaling and units.

## Results and Discussion

### QA/QC Results

To verify the accuracy of the applied analytical method in quantifying very low concentrations of PFCs expected in surface water samples, an experiment was performed. Briefly, different aliquots of the same water sample were spiked at different concentrations (0.2, 0.65, 2, 5, 10 and 20 ng L^−1^), extracted and injected. The tests were performed in sevenfold. The different concentration levels were chosen as follows: 0.2 ng L^−1^ and 0.65 ng L^−1^, respectively, for LOQ and environmental quality standard (EQS) of PFOS, and the concentrations between 5 and 20 ng L^−1^ represent PFCs environmental contamination levels, as reported previously (Nucci et al. 2019a and Nucci et al. 2019b). The concentration values chosen for the “low level” correspond to the LOQ values of the target analytes. The results obtained are shown in Table 3. In accordance with US EPA method 533, for analytes fortified at concentration ≤ 2 times LOQ level, the results with mean recovery ranging from 50 to 150% were acceptable. For analytes fortified at concentration > 2 times LOQ level, the acceptable recovery range is within 70–130% of the true value. All the obtained values, for the three concentration levels, are in line with the acceptance criteria set out in US EPA method 533, with mean percent recoveries ranging from 51 to 133% for low level, from 84 to 129% for medium level and from 84 to 113% for high level. The only exception is represented by PFODA; as given in Table 3; for high level, the mean percent recoveries are 57%. For this reason, the results for this compound are only semiquantitative for this study. The obtained recoveries were comparable or higher than those obtained by Juricova et al. (2022). The intraday relative standard deviation (RSD%) for low level ranged from 2 to 16%, from 0.2 to 25% for medium level and from 0.3% to 8% for high level. The R^2^ was greater than 0.999 for all the analytes under study, with the exception of PFHpA, PFOA, PFHpS and PFDS (Table 3). During the accuracy experiments and in every batch of river water samples, investigation of blank samples was also carried out to monitor background contamination. All the PFCs detected in the blank samples showed concentration levels less than a third of the corresponding LOQ, as indicated in the US EPA method 533.

**Table 3:** Linearity (*R*^2^), mean percent recovery (Mean %R) and relative standard deviation (RSD%) calculated at low level (0.20 ng L^−1^ for PFOS, 5.0 ng L^−1^ for PFOA and PFBA and 0.65 ng L^−1^ for all the other analytes), medium level (0.65 ng L^−1^ for PFOS, 10 ng L^−1^ for PFOA and PFBA and 2.0 ng L^−1^ for all the other analytes) and high level (2.0 ng L^−1^ for PFOS, 20 ng L^−1^ for PFOA and PFBA and 5.0 ng L^−1^ for all the other analytes)

	*R*^2^	Low level		Medium level		High level	
		Mean %R	RSD%	Mean %R	RSD%	Mean %R	RSD%
PFBA	0.999	100	3	84	0.2	84	0.3
PFPeA	0.999	94	11	117	7	90	2
PFBS	0.999	107	6	111	6	96	5
PFHxA	0.999	124	9	126	5	103	5
PFPeS	0.999	88	4	110	7	87	5
PFHpA	0.997	114	5	123	9	99	4
PFHxS	0.999	104	5	115	8	102	4
PFOA	0.997	114	3	86	2	92	3
PFHpS	0.998	93	7	113	8	93	3
PFNA	0.999	102	2	121	8	98	3
PFOS	0.999	133	2	104	5	113	7
PFNS	0.999	94	3	100	25	93	4
PFDA	0.999	95	2	114	6	89	2
PFDS	0.998	100	7	95	11	108	8
PFUdA	0.999	99	7	124	9	96	4
PFDoA	0.999	89	5	118	7	90	2
PFDoS	0.999	84	16	93	7	85	7
PFTrDA	0.999	103	9	129	9	112	8
PFTeA	0.999	94	5	122	5	103	5
PFHxDA	0.999	115	7	127	12	109	6
PFODA	0.999	51	6	99	10	57	11

**Fig. 3:**
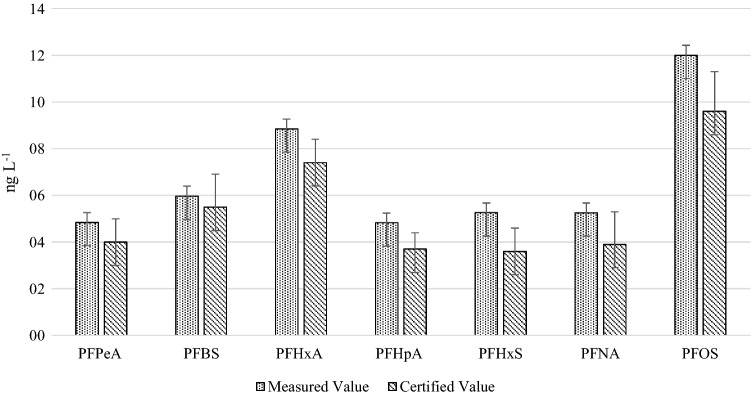
Comparison between measured and certified values (ng L^−1^). Uncertainty values in each group are expressed as error bars

The reliability of the applied method was also verified by analyzing the reference material IRMM-428 containing seven analytes (PFBS, PFHxS, PFOS, PFPeA, PFHxA, PFHpA and PFNA). The certified concentrations ranged from 3.6 to 9.6 ng L^−1^; the comparison between measured and certified values is presented in Fig. 3.

The good results achieved in the previously described experiments and in the analysis of the certified material make this method suitable for the analysis of PFCs in river water samples.

### PFCs in Freshwater Samples

**Fig. 4:**
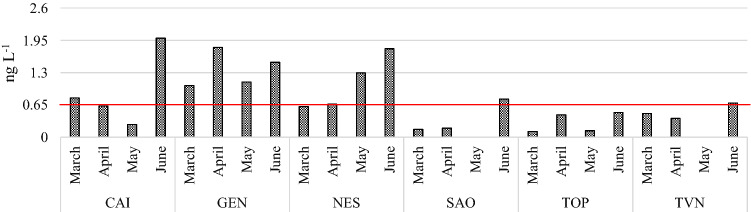
Concentration (ng L^−1^) of PFOS detected in river water samples. The red line represents the EQS for this compound

The results of the analysis of river water samples (*n* = 24) are summarized in Table 4. Target PFCs were detected in all the analyzed river water samples, but the levels of the analytes vary widely between months and sampling points. The sum of the 21 target analytes (∑_21_PFCs) detected in the analyzed river water samples ranged from 4.3 to 68.5 ng L^−1^ (Table 4). The concentrations of five of the six PFCs regulated by Italian 172/2015 Decree Law (i.e., 1000 ng L^−1^ for PFHxA, 3000 ng L^−1^ for PFPeA and PFBS, 7000 ng L^−1^ for PFBA, 100 ng L^−1^ for PFOA and 0.65 ng L^−1^ for PFOS) were below the regulatory limits in all the analyzed samples. On the other hand, PFOS exceeded the maximum concentration fixed by Italian 172/2015 Decree Law in 46% of the analyzed samples (Fig. 4, red line). For all the considered rivers, PFOS concentrations recorded in June were higher than the fixed EQS. The only exception was represented by TOP river, in which PFOS did not exceed the established EQS in any sampling month. On the contrary, GEN was the only river in which PFOS exceeded the fixed EQS in every sampling month (Fig. 4). Pignotti et al. (2017) and Zhu et al. (2015) reported a seasonal trend in which PFOS maximum concentration was recorded in winter and spring, respectively.

**Table 4:** Concentrations and sum (ng L^−1^) of 21 PFCs detected in river water samples in central Italy

	PFBA	PFPeA	PFBS	PFHxA	PFPeS	PFHpA	PFHxS	PFOA	PFHpS	PFNA	PFOS
*CAI*											
March	2.2	2.5	0.5	2.2	0.1	0.8	0.4	2.0	0.04	0.4	0.8
April	4.7	1.9	0.7	2.1	0.1	1.0	0.3	4.0	0.02	0.4	0.6
May	5.5	3.4	0.9	2.7	< LOD	1.2	0.04	1.8	0.6	< LOD	0.3
June	11.6	12.3	3.9	10.2	0.2	3.5	1.7	7.9	0.1	1.3	2.0
*GEN*											
March	3.0	4.4	0.7	3.9	0.1	1.4	0.8	2.7	0.04	0.4	1.0
April	6.1	7.5	1.5	6.5	0.2	3.1	0.7	7.1	0.1	0.9	1.8
May	11.0	6.5	1.5	5.6	0.02	2.1	0.8	4.0	0.6	0.1	1.1
June	14.5	12.3	2.6	12.4	nd	4.1	2.2	10	nd	nd	1.5
*NES*											
March	2.0	1.7	0.4	1.7	0.1	0.6	0.3	1.6	0.03	0.3	0.6
April	4.5	1.2	0.7	1.7	0.03	1.0	0.2	3.8	0.02	0.4	0.7
May	14.7	7.9	1.4	5.2	< LOD	2.3	0.6	4.9	0.8	0.4	1.3
June	19.2	10.5	2.3	12.4	0.2	6.5	1.1	11	0.1	2.2	1.8
*SAO*											
March	2.3	0.8	0.9	1.1	0.02	0.3	0.1	1.3	0.01	0.2	0.2
April	1.8	0.9	0.8	1.1	< LOD	0.4	0.1	3.5	0.01	0.2	0.2
May	3.6	1.7	1.7	1.1	< LOD	0.5	< LOD	0.4	0.6	< LOD	< LOD
June	9.7	4.6	4.5	4.8	0.1	1.4	0.3	5.0	0.05	0.7	0.8
*TOP*											
March	1.3	0.4	0.2	0.6	nd	0.2	0.1	0.8	0.01	0.1	0.1
April	2.2	< LOD	0.3	1.1	< LOD	0.4	0.1	3.3	0.1	0.3	0.5
May	10.3	4.2	0.3	1.6	< LOD	0.5	< LOD	1.8	0.6	< LOD	0.1
June	8.6	2.3	0.8	2.1	nd	0.6	0.1	3.1	0.04	0.3	0.5
*TVN*											
March	4.3	1.5	0.6	2.4	0.1	0.5	0.2	2.3	0.03	0.4	0.5
April	4.0	2.0	0.8	2.5	0.1	0.6	0.1	5.4	0.02	0.4	0.4
May	6.3	3.3	0.7	2.4	< LOD	0.8	< LOD	1.1	0.6	0.0	< LOD
June	6.9	5.1	1.3	3.6	0.05	0.8	0.2	3.1	0.03	0.4	0.7

	PFNS	PFDA	PFDS	PFUdA	PFDoA	PFDoS	PFTrDA	PFTeA	PFHxDA	PFODA	∑_21_PFCs
*CAI*											
March	0.1	0.4	< LOD	0.2	0.1	< LOD	0.1	0.1	< LOD	< LOD	12.9
April	0.05	0.3	< LOD	0.2	0.1	< LOD	0.2	0.1	< LOD	< LOD	16.6
May	0.3	0.4	< LOD	< LOD	< LOD	< LOD	< LOD	0.2	< LOD	< LOD	17.2
June	nd	1.2	nd	0.1	0.1	nd	0.1	< LOD	< LOD	< LOD	56.2
*GEN*											
March	< LOD	0.5	nd	0.1	0.1	< LOD	0.1	0.1	< LOD	< LOD	19.4
April	< LOD	0.7	< LOD	0.2	0.1	< LOD	0.1	0.1	< LOD	< LOD	36.6
May	0.3	0.6	< LOD	< LOD	< LOD	< LOD	< LOD	0.2	< LOD	< LOD	34.6
June	nd	nd	nd	nd	3.3	nd	nd	nd	nd	nd	63.0
*NES*											
March	< LOD	0.3	0.3	0.1	0.1	0.05	0.1	< LOD	< LOD	< LOD	10.0
April	< LOD	0.4	< LOD	0.1	0.1	< LOD	0.1	0.1	< LOD	0.2	15.3
May	< LOD	0.9	< LOD	< LOD	< LOD	< LOD	< LOD	0.3	< LOD	< LOD	40.6
June	nd	1.1	nd	0.1	0.1	nd	0.1	< LOD	< LOD	nd	68.5
*SAO*											
March	0.1	0.1	0.0	0.1	0.1	< LOD	0.1	0.1	< LOD	< LOD	7.8
April	< LOD	0.1	< LOD	0.1	< LOD	0.1	0.1	0.1	< LOD	0.2	9.7
May	0.3	0.0	< LOD	< LOD	< LOD	< LOD	< LOD	0.3	< LOD	< LOD	10.0
June	nd	0.7	nd	0.1	0.1	nd	0.1	0.1	< LOD	< LOD	33.0
*TOP*											
March	0.04	0.1	0.1	0.1	0.1	< LOD	0.1	0.1	< LOD	< LOD	4.3
April	0.1	0.2	0.1	0.3	0.2	0.1	0.2	0.2	0.3	0.2	10.3
May	0.3	0.2	< LOD	0.1	< LOD	< LOD	0.1	0.3	< LOD	< LOD	20.4
June	nd	0.4	nd	0.1	0.1	nd	< LOD	0.1	< LOD	nd	19.0
*TVN*											
March	0.1	0.3	0.1	0.2	0.1	0.05	0.1	0.1	< LOD	< LOD	13.7
April	0.1	0.2	< LOD	0.1	0.1	< LOD	0.1	0.1	0.2	< LOD	17.2
May	0.3	0.2	< LOD	< LOD	< LOD	< LOD	< LOD	0.3	< LOD	< LOD	15.9
June	nd	0.4	nd	< LOD	0.1	nd	0.1	< LOD	< LOD	nd	22.7

**Fig. 5:**
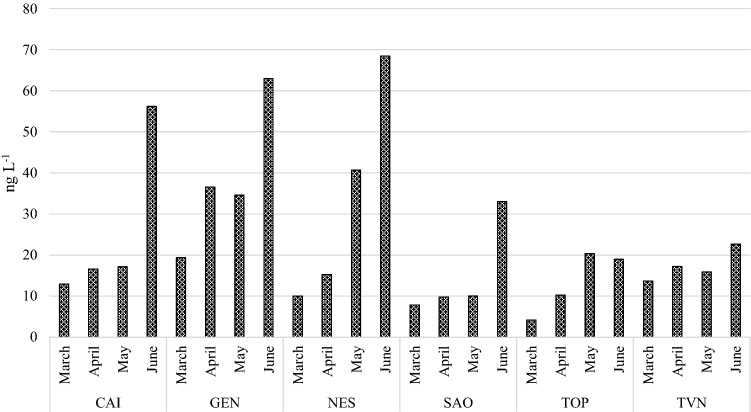
Monthly trend of the sum of the 21 investigated PFCs in the 6 studied rivers

PFOS concentration range found in this study varied from < LOD to 2.0 ng L^−1^. These values were consistent with PFOS concentrations found by Yamazaki et al. (2016) and Yang (2016) in Chinese river water samples and by Ahrens et al (2009) in German rivers, but much lower than those reported by Navarro et al. (2020) in Spanish river waters. Pignotti and Dinelli (2018) studied the distribution of PFOS in several rivers in north Italy, finding values comparable or higher than those reported in this study. Analyzing the individual PFCs contribution percentage, it appears that the short- and medium-chain PFCs (C4–C9) prevail over the long-chain PFCs (C10–C18). This is consistent with the trend found by Selvaraj et al. (2021) in Indian river waters and could be attributed to the increased use and higher solubility of short-chain PFCs compared to long-chain PFCs. The predominant congeners detected in this study were PFBA and PFPeA, followed by PFHxA and PFOA (Table 4). This result is in line with that reported by Zhu et al. (2015). Navarro et al. (2020), instead, reported PFOS as predominant compound, followed by PFOA, PFHxA and PFHxS. The ∑_21_PFCs in water samples of March, April, May and June ranged from 4.3 to 19.4 ng L^−1^, from 9.7 to 36.6 ng L^−1^, from 10.0 to 40.6 ng L^−1^ and from 19.0 to 68.5 ng L^−1^, respectively (Table 4 and Fig. 5). As shown in Fig. 5, the Σ_21_PFCs was much higher in June for all the studied rivers, except for TOP river in which the Σ_21_PFCs in May and June were comparable. The minimum Σ_21_PFCs found in this study (4.3 ng L^−1^) were comparable with that found by Zhu et al. (2015) in a Chinese river contaminated by several industrial waste but higher than that found by Navarro et al. (2020) in Spanish rivers. The maximum Σ_21_PFCs found in this study (47.3 ng L^−1^), instead, were much lower of those found by the same authors (Zhu et al. 2015), but higher than that found by Navarro et al. (2020) in Spanish rivers. Castiglioni et al. (2015) investigated the presence of PFCs in river water samples in north Italy, finding values 19 times higher than those reported in this study.

### Ecological Risk Assessment of PFCs

**Fig. 6:**
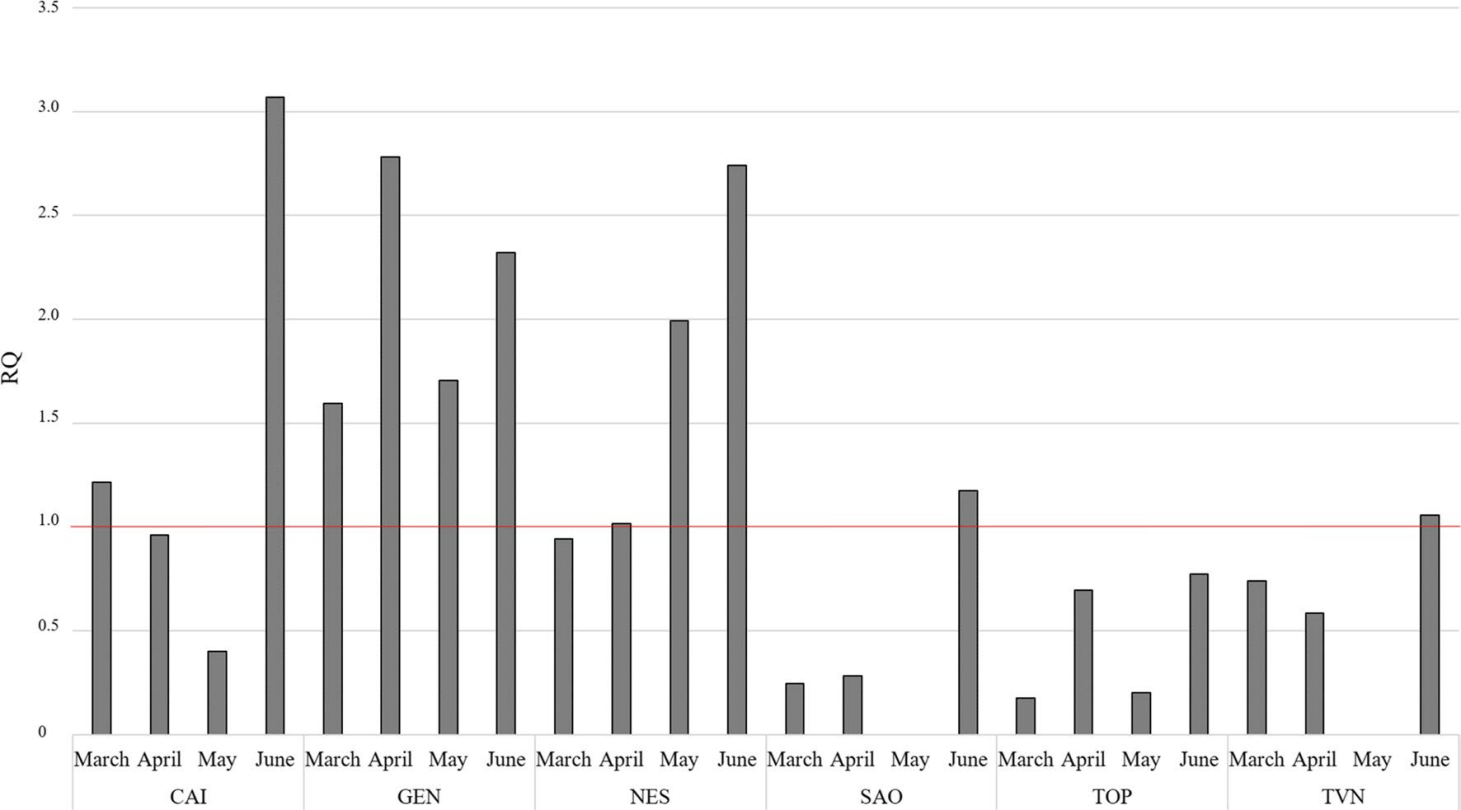
RQ values obtained for PFOS in the six river water samples in Umbria region (central Italy). RQ values above the red line indicate a high risk for aquatic ecosystem

In this work, the risk assessment for the aquatic biota relating to PFCs presence and levels in surface waters of Umbria region was conducted using a risk quotient (RQ) method conducted according to Lv et al. (2019). Briefly, RQ is determined throughout the ratio between the measured environmental concentration (ng L^−1^) and the corresponding EQS (ng L^−1^). When the RQ value is ≥ 1, it indicates that the risk of contamination in the area is high. When 0.1 ≤ RQ < 1 and 0.01 ≤ RQ < 0.1, it means that there is, respectively, medium and low risk of contamination in the aquatic environment (Yan et al. 2013). The RQ values, calculated for 6 PFCs (PFBA, PFPeA, PFBS, PFHxA, PFOA and PFOS) detected in river water samples of Umbria region, are reported in Table S1 (Supplementary Material). The RQ values were calculated only for six pollutants because the Italian 172/2015 Decree Law fixed the EQS only for these compounds (1000 ng L^−1^ for PFHxA, 3000 ng L^−1^ for PFPeA and PFBS, 7000 ng L^−1^ for PFBA and 100 ng L^−1^ for PFOA). For PFBA, PFPeA and PFBS, the calculated RQ values were much lower than 0.01 for all the investigated rivers and in all the sampling months (Table S1, Supplementary material). These values indicate a negligible risk for the aquatic organisms. For PFHxA, the calculated RQ ranged from 0.01 to 0.1 (low risk for the aquatic ecosystem) for three rivers (CAI, GEN and NES) in the month of June (Table S1, Supplementary material). Even if PFBA, PFPeA and PFHxA were some of the most abundant pollutants detected in river waters (Table 4), their lower ability to bioaccumulate in comparison to other monitored compounds causes them to have relatively high EQS values between 1000 and 7000 ng L^−1^ (Valsecchi et al. 2017) and, consequently, low RQ values. As regard PFOA, the calculated RQ highlighted a low risk for the aquatic organisms for almost all the considered rivers in all sampling months. The only exceptions were NES and CAI rivers in the month of June, where the calculated RQ was higher than 0.1 (medium risk). In the case of PFOS, the RQ values indicates a high risk for the aquatic ecosystem in 54% of the analyzed samples (Fig. 6). In the remaining 46% of the samples, the risk for the aquatic environment was classified as medium (Fig. 6).

### Potential Source Identification

**Fig. 7:**
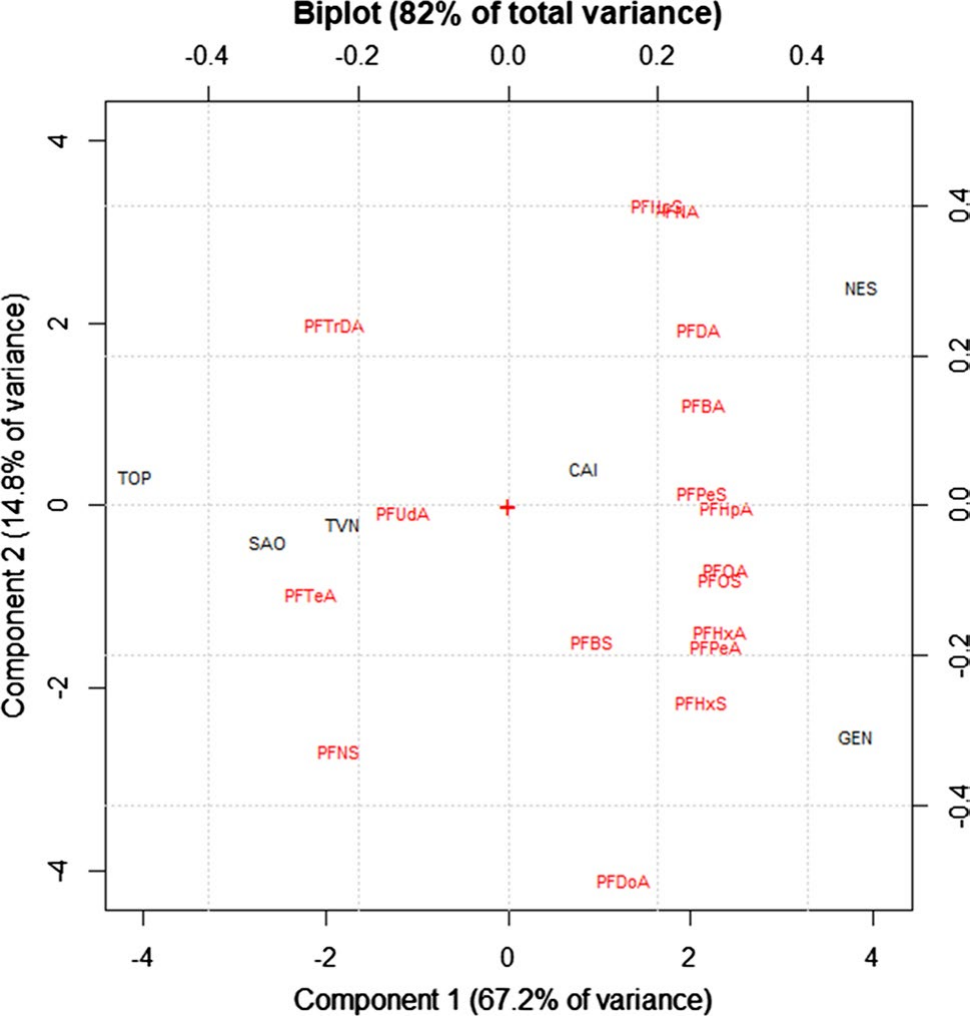
Biplot of the PCA (PC1 and PC2) performed on the concentration data of PFCs in river water samples

PCA results are summarized in the biplot reported in Fig. 7, while scores and loadings are shown in Tables 5 and 6, respectively. PFODA, PFHxDA, PFDoS and PFDS were excluded from the data analysis due to their low detection frequencies. Two significant components (PC1 and PC2), accounting for 85% of the total variance, were obtained. The biplot well separated two clusters of river water samples, each characterized by its emission profile (Fig. 7). The first cluster, in the left part of the biplot, consists of three river water samples (TVN, SAO and TOP) and four PFCs (PFTrDA, PFUdA, PFTeA and PFNS). All the previously mentioned rivers were affected by several industrial activities, including paper mills, cement plants and other smaller industry. Unfortunately, a comparison with the literature is difficult due to the lack of data; indeed, to our knowledge no study investigated the release of PFCs from cement plants. Kim et al. (2012) analyzed wastewater treatment plants from different industrial activities, including paper mill, founding a contamination profile different from that reported in this study and dominated by C6–C8 congeners. The second cluster, in the right part of the biplot, consists of three river water samples (NES, CAI and GEN) and several PFCs (Fig. 7). All the three river waters composing this cluster were affected by discharging of urban wastewater and runoff from agro-livestock farms. Tuan et al. (2021) analyzed water samples collected in rivers affected by agricultural production, animal husbandry and discharge of urban wastewaters finding high concentrations of short-chain PFCs (PFBA, PFPeA, PFHxS and PFHxA).

**Table 5:** Variance % and scores of the five components obtained by the PCA performed on the concentration data of PFCs

	PC1	PC2	PC3	PC4	PC5
% Variance	67.2	14.8	9.4	6.4	2.2
CAI	0.8237	0.4039	1.0749	− 1.8142	− 0.3281
GEN	3.8243	− 2.5266	0.3264	0.4551	0.1880
NES	3.8651	2.3951	− 0.4223	0.6147	0.2132
SAO	− 2.6233	− 0.3942	− 1.7943	− 0.6986	0.6276
TOP	− 4.0950	0.3242	1.6281	0.8107	0.3843
TVN	− 1.7948	− 0.2024	− 0.8127	0.6324	− 1.0851

**Table 6:** Loadings of the five components obtained by the PCA performed on the concentration data of PFCs

	PC1	PC2	PC3	PC4	PC5
PFBA	0.26227	0.136083	0.110094	0.350199	0.201391
PFPeA	0.276826	− 0.18869	0.138621	− 0.03741	− 0.08535
PFBS	0.112208	− 0.18161	− 0.44714	− 0.57479	0.502326
PFHxA	0.284135	− 0.16872	0.059316	0.010125	− 0.03076
PFPeS	0.261019	0.017013	0.042819	− 0.30195	− 0.55926
PFHpA	0.293049	− 0.00525	0.060588	0.035953	0.175176
PFHxS	0.258338	− 0.2613	0.177436	− 0.10826	0.085097
PFOA	0.290854	− 0.08611	0.041804	0.094958	0.079554
PFHpS	0.198601	0.39832	0.268973	0.138488	0.192961
PFNA	0.227796	0.394333	− 0.05693	− 0.09953	− 0.0069
PFOS	0.284755	− 0.0983	0.172497	− 0.02984	0.051073
PFNS	− 0.22712	− 0.32776	0.252412	− 0.17135	− 0.13508
PFDA	0.256022	0.236069	0.157806	− 0.25648	0.008543
PFUdA	− 0.13905	− 0.00963	0.621307	− 0.38804	− 0.01383
PFDoA	0.156145	− 0.49935	0.123002	0.211293	0.236037
PFTrDA	− 0.2306	0.241279	0.264416	− 0.23662	0.439815
PFTeA	− 0.26359	− 0.11744	0.247898	0.236518	0.185211

## Conclusions

The US EPA method 533 based on offline SPE of 250 mL of river water sample and subsequent analysis by HPLC–MS/MS was tested for 21 PFCs. The method performances, in terms of recovery, precision and sensibility, were satisfactory and in line with those established by US EPA. The LOQs ranged from 0.2 to 5 ng L^−1^ and allowed the detection of 21 PFCs in river water samples collected for four consecutive months in six rivers of Umbria region (central Italy). Most of the investigated substances were in line with the EQS established by Italian 172/2015 Decree Law, with the only exception of PFOS: 46% of the analyzed samples exceed the fixed EQS for this compound. PFBA and PFPeA followed by PFHxA and PFOA were the predominant compounds. The study of the monthly distribution of these pollutants highlighted that the ∑_21_PFCS was lower in March and increased toward the summer months for all the investigated rivers. The ecological risk assessment, based on the calculation of RQ, highlighted that the risk for aquatic environments for all the studied rivers for PFBA, PFPeA, PFBS, PFHxA and PFOA was low or negligible. Only for PFOA, a medium risk for NES and GEN rivers in the month of June was determined. As regard PFOS, a high risk for the aquatic environment was found in 54% of the river water samples, with RQ values ranging from 1.0 to 3.1. For the remaining 46% of the samples, the risk was medium, with RQ values from 0.2 to 0.96. This investigation confirmed the extensive diffusion of PFCs in all the analyzed river waters and the importance of PFCs determination even in areas where there are no direct emission sources over the territory. The obtained data may improve the knowledge of the occurrence and diffusion of PFCs in the aquatic environment and help assess their potential risk. The concentration data may also be used to build a database useful for estimating the background pollution in the rivers of central Italy.

## Supplementary Information

Below is the link to the electronic supplementary material.

Supplementary file1 (DOCX 15 kb)

## References

[CR1] Ahmed MB, Johir MAH, McLaughlan R, Nguyen LN, Xu B, Nghiem LD (2020). Per- and polyfluoroalkyl substances in soil and sediments: occurrence, fate, remediation and future outlook. Sci Total Environ.

[CR2] Ahrens L, Plassmann M, Xie ZY, Ebinghaus R (2009). Determination of polyfluoroalkyl compounds in water and suspended particulate matter in the river Elbe and North Sea, Germany. Front Environ Sci Eng.

[CR3] Ayanda IO, Yang M, Yu Z, Zha J (2018). Cytotoxic and genotoxic effects of perfluorododecanoic acid (PFDoA) in Japanese medaka. Knowl Manag Aquat Ecosyst.

[CR4] Bach CC, Henriksen TB, Bossi R, Bech BH, Fuglsang J, Olsen J, Nohr EA (2015). Perfluoroalkyl acid concentrations in blood samples subjected to transportation and processing delay. PLoS ONE.

[CR5] Barreca S, Busetto M, Vitelli M, Colzani L, Clerici L, Dellavedova P (2018). Online solid-phase extraction LC-MS/MS: A rapid and valid method for the determination of perfluorinated compounds at sub ng·L^−1^ level in natural water. J Chem.

[CR6] Berger U, Kaiser MA, Kärrman A, Barber JL, van Leeuwen SPJ (2011). Recent developments in trace analysis of poly- and perfluoroalkyl substances. Anal Bioanal Chem.

[CR7] Blaine A, Rich C, Hundal L, Lau CS, Mills M, Harris K, Higgins CP (2013). Uptake of perfluoroalkyl acids into edible crops via land applied biosolids: field and greenhouse studies. Environ Sci Technol.

[CR8] Boisvert G, Sonne C, Rigét FF, Dietz R, Letcher RJ (2019). Bioaccumulation and biomagnification of perfluoroalkyl acids and precursors in east Greenland polar bears and their ringed seal prey. Environ Pollut.

[CR9] Bonelli MG, Brambilla GF, Manni A (2020) Spatial distribution and sources of total chromium and perfluoroalkyl substances (PFAS) in Northern Italy rivers. In: 2nd International conference on resources and environment sciences. 10.1088/1755-1315/563/1/012019

[CR10] Butt CM, Berger U, Bossi R, Tomy GT (2010). Levels and trends of poly- and perfluorinated compounds in the arctic environment. Sci Total Environ.

[CR11] Campo J, Lorenzo M, Pérez F, Picó Y, Farré M, Barceló D (2016). Analysis of the presence of perfluoroalkyl substances in water, sediment and biota of the Jucar River (E Spain). Sources, partitioning and relationships with water physical characteristics. Environ Res.

[CR12] Castiglioni S, Valsecchi S, Polesello S, Rusconi M, Melis M, Palmiotto M, Manenti A, Davoli E, Zuccato E (2015). Sources and fate of perfluorinated compounds in the aqueous environment and in drinking water of a highly urbanized and industrialized area in Italy. J Hazard Mater.

[CR13] Charavgis F, Cingolani A, Renzi S (2022). Il monitoraggio delle sostanze perfluoroalchiliche nelle acque superficiali e sotterranee della regione Umbria (2018–2021).

[CR14] Chiesa LM, Pavlovic R, Arioli F, Nobile M, Di Cesare F, Mosconi G, Falletta E, Malandra R, Panseri S (2022). Presence of perfluoroalkyl substances in Mediterranean Sea and North Italian lake fish addressed to Italian consumer. Int J Food Sci.

[CR15] Commission directive 2009/90/EC of 31 July 2009 laying down, pursuant to Directive 2000/60/EC of the European Parliament and of the Council, technical specifications for chemical analysis and monitoring of water status

[CR16] Decreto legislativo 13 ottobre 2015, n. 172. Attuazione della direttiva 2013/39/UE, che modifica le direttive 2000/60/CE per quanto riguarda le sostanze prioritarie nel settore della politica delle acque

[CR17] Deng H, Wang H, Liang M, Su X (2019). A novel approach based on supramolecular solvent microextraction and UPLC-Q-Orbitrap HRMS for simultaneous analysis of perfluorinated compounds and fluorine-containing pesticides in drinking and environmental water. Microchem J.

[CR18] Domingo JL (2012). Health risks of dietary exposure to perfluorinated compounds. Environ Int.

[CR19] European Commission (EC) (2013) Directive 2013/39/EU of the European parliament and of the council of 12 August 2013 amending Directives 2000/60/EC and 2008/105/EC as regards priority substances in the field of water policy. Official Journal of the European Union. L 226/1

[CR20] Fauconier G, Groffen T, Wepener V, Bervoets L (2020). Perfluorinated compounds in the aquatic food chains of two subtropical estuaries. Sci Total Environ.

[CR21] European Commission (EC) (2020) SANTE/12682/2019: analytical quality control and method validation procedures for pesticide residues analysis in food and feed. https://www.eurl-pesticides.eu/userfiles/file/EurlALL/AqcGuidance_SANTE_2019_12682.pdf. Accessed July 2021

[CR22] González-Barreiro C, Martínez-Carballo E, Sitka A, Scharf S, Gans O (2006). Method optimization for determination of selected perfluorinated alkylated substances in water samples. Anal Bioanal Chem.

[CR23] Jurikova M, Dvorakova D, Pulkrabova J (2022). The occurrence of perfluoroalkyl substances (PFAS) in drinking water in the Czech Republic: a pilot study. Environ Sci Pollut Res Int.

[CR24] Kancharla S, Alexandridis P, Tsianou M (2022). Sequestration of per- and polyfluoroalkyl substances (PFAS) by adsorption: Surfactant and surface aspects. Curr Opin Colloid Interface.

[CR25] Kim SK, Im JK, Kang YM, Jung SY, Kho YL, Zoh KD (2012). Wastewater treatment plants (WWTPs)-derived national discharge loads of perfluorinated compounds (PFCs). J Hazard Mater.

[CR26] Kim M, Park MS, Son J, Park I, Lee H, Kim C, Min B, Ryoo J, Choi KS, Lee D, Lee HS (2015). Perfluoroheptanoic acid affects amphibian embryogenesis by inducing the phosphorylation of ERK and JNK. Int J Mol Med.

[CR27] Kurwadkar S, Dane J, Kanel SR, Nadagouda MN, Cawdrey RW, Ambade B, Struckhoff GC, Wilkin R (2022). Per- and polyfluoroalkyl substances in water and wastewater: a critical review of their global occurrence and distribution. Sci Total Environ.

[CR28] Lam NH, Cho CR, Kannan K, Cho HS (2017). A nationwide survey of perfluorinated alkyl substances in waters, sediment and biota collected from aquatic environment in Vietnam: distributions and bioconcentration profiles. J Hazard Mater.

[CR29] Latała A, Nędzi M, Stepnowski P (2009). Acute toxicity assessment of perfluorinated carboxylic acids towards the Baltic microalgae. Environ Toxicol Pharmacol.

[CR30] Li J, Guo F, Wang Y, Liu J, Cai Z, Zhang J, Zhao Y, Wu Y (2012). Development of extraction methods for the analysis of perfluorinated compounds in human hair and nail by high performance liquid chromatography tandem mass spectrometry. J Chromatogr A.

[CR31] Li J, Guo F, Wang Y, Zhang J, Zhong Y, Zhao Y, Wu Y (2013). Can nail, hair and urine be used for biomonitoring of human exposure to perfluorooctane sulfonate and perfluorooctanoic acid?. Environ Int.

[CR32] Liang L, Pan Y, Bin L, Liu Y, Huang W, Li R, Lai KP (2022). Immunotoxicity mechanisms of perfluorinated compounds PFOA and PFOS. Chemosphere.

[CR33] Lindstrom AB, Strynar MJ, Libelo EL (2011). Polyfluorinated compounds: past, present, and future. Environ Sci Technol.

[CR34] Liu C, Chang VW, Gin KY, Nguyen VT (2014). Genotoxicity of perfluorinated chemicals (PFCs) to the green mussel (*Perna viridis*). Sci Total Environ.

[CR35] Lv J, Guo C, Liang S, Zhang Y, Xu J (2019). Partitioning behavior, source identification, and risk assessment of perfluorinated compounds in an industry-influenced river. Environ Sci Eur.

[CR36] Miralles-Marco A, Harrad S (2015). Perfluorooctane sulfonate: a review of human exposure, biomonitoring and the environmental forensics utility of its chirality and isomer distribution. Environ Int.

[CR37] Navarro I, De la Torre A, Sanz P, Martínez MLÁ (2020). Perfluoroalkyl acids (PFAAs): distribution, trends and aquatic ecological risk assessment in surface water from Tagus River basin (Spain). Environ Pollut.

[CR38] Nucci M, Cingolani A, Charavgis F, Renzi S (2019). Le sostanze perfluoroalchiliche (PFAS) in Umbria: stato attuale e programmi di monitoraggio.

[CR39] Nucci M, Cingolani A, Charavgis F, Renzi S (2019). I PFAS in Umbria nel 2018 - monitoraggio delle sostanze perfluoroalchiliche su acque superficiali, acque sotterranee e scarichi in Umbria.

[CR40] Ojemaye CY, Petrik L (2019). Occurrences, levels and risk assessment studies of emerging pollutants (pharmaceuticals, perfluoroalkyl and endocrine disrupting compounds) in fish samples from Kalk Bay harbour, South Africa. Environ Pollut.

[CR41] Organization for Economic Co-operation and Development (2018) Toward a new comprehensive global database of per- and polyfluoroalkyl substances (PFASs): summary report on updating the OECD 2007 List of Per- and Polyfluoroalkyl SubstancES (PFASs)

[CR42] Pignotti E, Casas G, Llorca M, Tellbüscher A, Almeida D, Dinelli E, Farré M, Barceló D (2017). Seasonal variations in the occurrence of perfluoroalkyl substances in water, sediment and fish samples from Ebro Delta (Catalonia, Spain). Sci Total Environ.

[CR43] Pignotti E, Dinelli E (2018). Distribution and partition of endocrine disrupting compounds in water and sediment: case study of the Romagna area (North Italy). J Geochem Explor.

[CR44] Post GB, Cohn PD, Cooper KR (2012). Perfluorooctanoic acid (PFOA), an emerging drinking water contaminant: a critical review of recent literature. Environ Res.

[CR45] Savoca D, Pace A (2021). Bioaccumulation, biodistribution, toxicology and biomonitoring of organofluorine compounds in aquatic organisms. Int J Mol Sci.

[CR46] Sedlak MD, Benskin JP, Wong A, Grace R, Greig DJ (2017). Per- and polyfluoroalkyl substances (PFASs) in San Francisco Bay wildlife: temporal trends, exposure pathways, and notable presence of precursor compounds. Chemosphere.

[CR100] Selvaraj KK, Murugasamy M, Nikhil NP, Elaiyaraja A, Sampath S, Krishnamoorthi V, He H, Ramaswamy BR (2021) Investigation of distribution, sources and flux of perfluorinated compounds in major southern Indian rivers and their risk assessment. Chemosphere 277. 10.1016/j.chemosphere.2021.130228 10.1016/j.chemosphere.2021.130228 34384168

[CR47] Stubleski J, Salihovic S, Lind L, Lind PM, van Bavel B, Kärrman A (2016). Changes in serum levels of perfluoroalkyl substances during a 10-year follow-up period in a large population-based cohort. Environ Int.

[CR48] Sungur S (2022) Chapter 9—Environmental fate and transportation of perfluorinated compounds. In: Emerging contaminants in the environment, pp 203–224. 10.1016/B978-0-323-85160-2.00017-2

[CR49] Thompson J, Eaglesham G, Mueller J (2011). Concentrations of PFOS, PFOA and other perfluorinated alkyl acids in Australian drinking water. Chemosphere.

[CR50] Tuan DH, Anh PTL, Lam BN (2021). Distribution of perfluoroalkyl substances (PFASs) in the water of the Bac Hung Hai River, Van Giang district, Hung Yen province, Vietnam. VN J Hydrometeorol.

[CR51] UNEP (United Nations Environment Program) (2009) Decision SC-4/17: listing of PFOS, its salts and perfluorooctane sulfonyl fluoride (PFOSF) in Annex B to the Stockholm Convention. UN Environment (UNEP), Secretariat of the Basel, Rotterdam and Stockholm Conventions, Geneva, Switzerland

[CR52] UNEP (United Nations Environment Program) (2019) Decision SC-9/12: listing of Perfluorooctanoic acid (2019), its salts and PFOA-related compounds. UN Environment (UNEP), Secretariat of the Basel, Rotterdam and Stockholm Conventions, Geneva, Switzerland

[CR53] US EPA (2010) United States Environmental Protection Agency. Office of Pollution Prevention and Toxics. 2010/2015 PFOA stewardship program. http://www.epa.gov/opptintr/pfoa/pubs/stewardship/index.html. Last updated April 29, 2010

[CR54] US EPA (2012) United States Environmental Protection Agency. Perfluorooctanoic acid (PFOA) and fluorinated telomers 2011 Annual Progress Reports. Posted February 6, 2012. http://www.epa.gov/oppt/pfoa/pubs/stewardship/preports5.html

[CR55] US EPA (United States Environmental Protection Agency) Method 533 (2019) Method 533: determination of per- and polyfluoroalkyl substances in drinking water by isotope dilution anion exchange solid phase extraction and liquid chromatography/tandem mass spectrometry

[CR56] US EPA (United States Environmental Protection Agency) Method 1633 (2022) Analysis of per- and polyfluoroalkyl substances (PFAS) in aqueous, solid, biosolids, and tissue samples by LC-MS/MS

[CR57] Valsecchi S, Rusconi M, Mazzoni M, Viviano G, Pagnotta R, Zaghi C, Serrini G, Polesello S (2015). Occurrence and sources of perfluoroalkyl acids in Italian river basins. Chemosphere.

[CR58] Valsecchi S, Conti D, Crebelli R, Polesello S, Rusconi M, Mazzoni M, Preziosi E, Carere M, Lucentini L, Ferretti E, Balzamo S, Simeone MG, Aste F (2017). Deriving environmental quality standards for perfluorooctanoic acid (PFOA) and related short chain perfluorinated alkyl acids. J Hazard Mater.

[CR59] Wang T, Wang YW, Liao CY, Cai YQ, Jiang GB (2009). Perspectives on the inclusion of perfluorooctane sulfonate into the Stockholm convention on persistent organic pollutants. Environ Sci Technol.

[CR60] Wang Z, Dewitt JC, Higgins CP, Cousins IT (2017). A never-ending story of per- and polyfluoroalkyl substances (PFASs)?. Environ Sci Technol.

[CR61] Yamazaki E, Falandysz J, Taniyasu S, Hui G, Jurkiewicz G, Yamashita N, Yang YL, Lam PKS (2016). Perfluorinated carboxylic and sulphonic acids in surface water media from the regions of Tibetan Plateau: indirect evidence on photochemical degradation. J Environ Sci Health A Tox Hazard Subst Environ Eng.

[CR62] Yan C, Yang Y, Zhou J, Liu M, Nie M, Shi H, Gu L (2013). Antibiotics in the surface water of the Yangtze Estuary: occurrence, distribution and risk assessment. Environ Pollut.

[CR63] Yang YL (2016) Distribution and source analysis of typical perfluorinated compounds in the eastern China and the Grand canal. A thesis submitted to Chinese Academy of Geological Sciences for the Doctor degree of Geochemistry

[CR64] Zhang T, Sun H, Qin X, Gan Z, Kannan K (2014). PFOS and PFOA in paired urine and blood from general adults and pregnant women: assessment of urinary elimination. Environ Sci Pollut Res.

[CR65] Zhang G, Pan Z, Wu Y, Shang R, Zhou X, Fan Y (2019). Distribution of perfluorinated compounds in surface water and soil in partial areas of Shandong province, China. Soil Sediment Contam.

[CR66] Zhang F, Wang Y, Wei Z, Zhang G, Wang J (2021). Perfluorinated compounds in a river basin from Qing Hai-Tibet plateau: occurrence, sources and key factors. Ecotoxicol Environ Saf.

[CR67] Zhang Y, Qv Z, Wang J, Yang Y, Chen X, Wang J, Zhang Y, Zhu L (2022). Natural biofilm as a potential integrative sample for evaluating the contamination and impacts of PFAS on aquatic ecosystems. Water Res.

[CR68] Zhu Z, Wang T, Meng J, Wang P, Li Q, Lu Y (2015). Perfluoroalkyl substances in the Daling River with concentrated fluorine industries in China: seasonal variation, mass flow, and risk assessment. Environ Sci Pollut Res.

