# Supplementary material for: Perfluorinated Compounds (PFCs) in River Waters of Central Italy: Monthly Variation and Ecological Risk Assessment (ERA)
Source: Arch Environ Contam Toxicol. 2023 Apr 6;84(3):332–46. doi: 10.1007/s00244-023-00993-4 (PMC10130131; doi:10.1007/s00244-023-00993-4)
Supplement: Supplementary file 1 — Supplementary file1 (DOCX 15 kb) [file 244_2023_993_MOESM1_ESM.docx]

**Perfluorinated Compounds (PFCs) in river waters of central Italy: monthly variation and Ecological Risk Assessment (ERA)**

Federica Castellani^a,b^, Mara Galletti^c^, Fedra Charavgis^d^, Alessandra Cingolani^d^, Sonia Renzi^d^, Mirko Nucci^d^, Carmela Protano^a^, Matteo Vitali^a,*^

^a^ Department of Public Health and Infectious Diseases, University of Rome La Sapienza, P.le Aldo Moro, 5, 00185 Rome, Italy

^b^ Department of Ecological and Biological Sciences, Tuscia University, Largo dell’Università snc, 01100 Viterbo, Italy

^c^ ARPA Umbria, Via Carlo Alberto dalla Chiesa, 23, 05100 Terni, Italy

^d^ ARPA Umbria, Via Pievaiola 207/B-3, 06132 Perugia, Italy

* corresponding Author

**S1** Table 5

**Table 5.** EQS (ng L^-1^) and obtained RQ for six PFCs detected in six river water samples in central Italy.

|  |  | **PFBA** | **PFPeA** | **PFBS** | **PFHxA** | **PFOA** | **PFOS** |
| --- | --- | --- | --- | --- | --- | --- | --- |
|  | **EQS** | **7000** | **3000** | **3000** | **1000** | **100** | **0.65** |
| CAI | **March** | 0.0003 | 0.0008 | 0.0002 | 0.0022 | 0.02 | 1.2 |
|  | **April** | 0.0007 | 0.0006 | 0.0002 | 0.0021 | 0.04 | 1.0 |
|  | **May** | 0.0008 | 0.0011 | 0.0003 | 0.0027 | 0.02 | 0.4 |
|  | **June** | 0.0017 | 0.0041 | 0.0013 | 0.0102 | 0.08 | 3.1 |
| GEN | **March** | 0.0004 | 0.0015 | 0.0002 | 0.0039 | 0.03 | 1.6 |
|  | **April** | 0.0009 | 0.0025 | 0.0005 | 0.0065 | 0.07 | 2.8 |
|  | **May** | 0.0016 | 0.0022 | 0.0005 | 0.0056 | 0.04 | 1.7 |
|  | **June** | 0.0021 | 0.0041 | 0.0009 | 0.0124 | 0.10 | 2.3 |
| NES | **March** | 0.0003 | 0.0006 | 0.0001 | 0.0017 | 0.02 | 0.9 |
|  | **April** | 0.0006 | 0.0004 | 0.0002 | 0.0017 | 0.04 | 1.0 |
|  | **May** | 0.0021 | 0.0026 | 0.0005 | 0.0052 | 0.05 | 2.0 |
|  | **June** | 0.0027 | 0.0035 | 0.0008 | 0.0124 | 0.11 | 2.7 |
| SAO | **March** | 0.0003 | 0.0003 | 0.0003 | 0.0011 | 0.01 | 0.2 |
|  | **April** | 0.0003 | 0.0003 | 0.0003 | 0.0011 | 0.04 | 0.3 |
|  | **May** | 0.0005 | 0.0006 | 0.0006 | 0.0011 | 0.004 | - |
|  | **June** | 0.0014 | 0.0015 | 0.0015 | 0.0048 | 0.05 | 1.2 |
| TOP | **March** | 0.0002 | 0.0001 | 0.0001 | 0.0006 | 0.01 | 0.2 |
|  | **April** | 0.0003 | - | 0.0001 | 0.0011 | 0.03 | 0.7 |
|  | **May** | 0.0015 | 0.0014 | 0.0001 | 0.0016 | 0.02 | 0.2 |
|  | **June** | 0.0012 | 0.0008 | 0.0003 | 0.0021 | 0.03 | 0.8 |
| TVN | **March** | 0.0006 | 0.0005 | 0.0002 | 0.0024 | 0.02 | 0.7 |
|  | **April** | 0.0006 | 0.0007 | 0.0003 | 0.0025 | 0.05 | 0.6 |
|  | **May** | 0.0009 | 0.0011 | 0.0002 | 0.0024 | 0.01 | - |
|  | **June** | 0.0010 | 0.0017 | 0.0004 | 0.0036 | 0.03 | 1.1 |
